# Book Reviews

**Published:** 1825-06

**Authors:** 


### CRITICAL ANALYSIS
OF
### ENGLISH AND FOREIGN LITERATURE,
RELATIVE TO THE VARIOUS BRANCHES OF
### Medical Science.

* Quæ laudanda forent, et quæ culpanda, vicissim

Illa, prius, cretâ; mox hæc, carbone, *notamus*.—PERSIUS.

### DIVISION I.
### ENGLISH.

ART. I.—*An Account of the Disease lately prevalent at the General Penitentiary.* By P. MERE LATHAM, M.D. Fellow of the Royal College of Physicians, and Physician to St. Bartholomew's Hospital. —8vo. pp. 286. T. and G. Underwood, London, 1825.

ART. II.—*Third Vindication of the General Penitentiary; showing that there is no Ground whatever for supposing that the Situation of that Prison had any Share in producing the late Disease among the Prisoners confined there. Being an Answer to some Observations contained in a Work published by* P. MERE LATHAM, M.D. *entitled "An Account of the Disease lately prevalent at the General Penitentiary."* By GEORGE HOLFORD, M.P.—8vo. pp. 155. Rivington, London, 1825.

WE have often professed ourselves to be the most impartial critics in the world, and we trust that by this time our readers are disposed to give us credit for some virtue in this way. On the present occasion, however, we feel that our reputation runs some hazard as we approach a discussion, in which personal feelings and contending interests have been so mixed up as to leave it extremely difficult to get at the real merits of the question. Neither can we feel quite satisfied that we ourselves may not have been influenced, to a certain extent, by the numerous reports which have been in circulation; and thus unconsciously have had our minds imbued with prejudices which would detract from the correctness of our judgment and the fairness of our criticisms. In this dilemma, we have heretofore, and still purpose to avoid altogether entering into the *disputes* (we cannot use a milder term,) which have occurred between the Committee of the Penitentiary and all their medical officers successively; and shall restrict ourselves to a history of the disease, stating the evidence on both sides, wherever a difference of opinion has existed.

The work before us is entitled "An Account of the Disease lately prevalent at the General Penitentiary," and it is in this point of view alone that we shall regard it;—in this point of view alone it can become generally interesting, or really valuable to the profession at large. It is the first account of this epidemic which has been given,—or at least published; and if we have not entered upon the subject before, we repeat it is simply because we have regarded the pages of a Medical Journal as unfit for the discussion of any but medical topics. But to the point.

Dr. LATHAM commenced his attendance at the Penitentiary in March 1823, and continued it till May 1824, a period of fifteen months, during which he informs us that he made memoranda of all the circumstances which appeared to be of importance: from the memoranda, thus taken on the spot, it is that the present work is compiled.

The history of the disease is given under various heads, according to the nature of the symptoms,—viz. Scurvy, Bowel Complaints, Disorders of the Brain and Nervous System, Fever, and Intercurrent Diseases; after which we have a review of the extent of the disease at different periods; an account of the influence produced upon the health of the prisoners by their removal from the Penitentiary; and, lastly, we have an inquiry into the origin of the disease. Of each of these subjects we shall give a short account, and one almost purely analytic.

*The Scurvy.*—On the first visit made by Dr. Latham and his colleague (Dr. Roget) to the Penitentiary, the condition of the skin, as it existed in almost every prisoner, attracted particular notice. It presented the appearance commonly called goose-skin, was dry and without perspiration, the cuticle falling off in the state of minute scales or powder. This condition, the author observes, generally prevails when any considerable debility is present: in the Penitentiary, "it was found almost in every prisoner to a very remarkable degree." Associated with this peculiar condition of the skin, there were blue or livid spots, formed by ecchymosis, and varying in size; some were no bigger than pin's heads, some as large as peas, and others still larger, and of less regular shape, apparently formed by the blending together of several smaller ones. These were distributed over every part of the extremities; and in some instances the arms, legs, and buttocks, were almost uniformly livid; and, lastly, with respect to this symptom, the conjunctiva and upper eye-lid were occasionally affected with a similar ecchymosis. The gums were soft and spongy: in one man they were "in a state of absolute rottenness, the gums bleeding and broken down, the teeth loose, and their fangs half exposed." The affection of the gums, however, appears to have borne a distinct relation to that of the skin. In many the muscles of the leg were hard and rigid; in a few the extremities were œdematous.

The scurvy, however, constituted but a very inconsiderable part of the malady, and one which speedily gave way to the remedies employed; having diminished very much during the first month, and being almost entirely gone by the end of the second. The treatment consisted in a change of diet, by substituting four ounces of flesh meat in place of pease and barley soup for dinner, white bread for brown, and the addition of rice and three oranges a-day to each individual.

*Bowel Complaints.*—These were very severe, very general in their occurrence, and so varied in their symptoms, as to render it impossible to class them under any of the usual denominations of diarrhœa, dysentery, and cholera: "there was every degree and species of flux that was ever seen or described."

"There were cases which corresponded with the descriptions of the Indian cholera. The patients were seized with intolerable cramps at the pit of the stomach. They retched and vomited, and a thin turbid serum ran from their bowels, followed by severe tenesmus. The pulse became feeble and frequent; they were pale and chilly, and a sudden anguish pervaded the whole frame. Again, there were cases which corresponded with the common autumnal cholera of this country. The patients had severe griping of the intestines generally, and cramps of the extremities; while pure and unmixed bile ran from the bowels, scalding them (as they expressed it) like melted lead in its passage.

"Moreover, there was every kind and degree of dysentery: some purged pure blood in large quantities; others a fluid like the water in which raw flesh had been washed. In some the evacuations, otherwise healthy, were just streaked with blood; in some they contained (what seemed to be) lumps of flesh. In others they were mixed with mucus and slime, or they consisted of mucus and slime altogether.

"Again, there were cases which differed very little from the diarrhœa of common casual occurrence, except that they were quite intractable by common remedies. The evacuations were loose and attended with griping, but feculent and without any morbid quality.

"Lastly, there were cases which had no resemblance whatever, either to cholera, or dysentery, or diarrhœa, or to any disorder that has obtained a name. In the evacuations there appeared nothing that had any sensible quality of fæces, of bile, or blood, or (of what is understood by) mucus and slime. But they consisted sometimes of a mass, like green or black grapes in a state of fermentation; *sometimes of a matter like yeast; sometimes they were, in colour and consistence, like half-slaked lime, when it is beginning to crumble ; and sometimes like a thin mixture of chalk and water, and always intolerably sour and offensive, and in enormous quantity.*" (P. 32—34.)

This last variety of flux is certainly of rare occurrence, but not so rare as Dr. Latham seems to have supposed ; at least, it has been both described and received a name. It is the *diarrhœa gypsata* of Dr. Mason Good, and is thus described by Dr. Baillie, in the Transactions of the College of Physicians, vol. v.—"*The evacuation consists of a matter resembling in its appearance a mixture of water and lime, which is generally very frothy on its surface. When the disease is violent, the discharges are copious and very numerous, of a pale colour and sour smell, and the froth looks like yeast.* When it changes to a milder form, the evacuations are still more or less pale, but of the consistence of pudding, and do not occur oftener than two or three times in twenty-four hours. The appetite is often good, but sometimes defective. The countenance thin and sallow, but not much emaciated. The pulse varies but little from the standard of health, but is rather disposed to acceleration. The tongue is generally covered with a white pus, of moderate thickness. The urine of a somewhat deeper hue than natural, generally clear, occasionally turbid. An examination of the abdomen discovers nothing unnatural. The bowels are apt to be distended with wind, but there is no tumor or sense of pain upon pressure." If the reader casts his eye once more over the description of the last variety of flux, as given by Dr. Latham, (particularly the portion which we have put in italics,) he will perceive that the resemblance to the species of diarrhœa described by Dr. Baillie is so complete, as to leave no doubt of their identity. The descriptions further coincide in the circumstances of the disease being apt to recur after an apparent cure, and to be aggravated by mental depression.

To return to the author before us. Little or no connexion could be traced between the nature of the flux and the degree of danger : those who had simple diarrhœa sinking under the complaint as frequently as those affected with the more severe forms of cholera or dysentery ; and hence assistance in forming the prognosis was sought for from other symptoms. The abdomen was for the most part partially, and in some universally, distended and tympanitic. This was more remarkable among the women than the men; and some of the former continued, we are told, for months afterwards, with the abdomen "enormously prominent." In many of both sexes, the belly remained natural throughout; and in some it was collapsed and retracted. In one case only was any distinct tumor perceptible: it arose from enlargement of the liver. The abdominal pain varied in different individuals: the great majority had constant uneasiness in the bowels; some only suffered immediately before each evacuation, some immediately after. But one of the most frequent and distressing complaints was a "sinking at the pit of the stomach." This peculiar and depressing sensation is often present in severe cases of dyspepsia, particularly in those who have accustomed themselves to the use of spirituous liquors, and is generally described as more intolerable to bear than actual pain. "When we were interrogating them upon circumstances apparently more urgent," says Dr. Latham, "they would interrupt us, and exclaim, 'But this sinking—this sinking: pray do something for this sinking!'"

Many had violent paroxysms of pain, which occupied different parts of the abdomen; they resembled colic, and pressure, equally applied over a large surface, gave relief during the paroxysm, but aggravated the pain after this had subsided. The tongue was clean, moist, and natural, in the great majority of cases, whether the disease assumed the character of cholera, dysentery, or diarrhœa; in a few cases it was rather redder than natural; but in none was there the red, glossy, smooth tongue of dysentery. The pulse, in the majority, had no morbid character sufficient to warrant bleeding; in a few, however, this or some other depletion was deemed necessary to reduce it. In many it was entirely natural.

"Such was the remarkable diversity of symptoms which accompanied this strange and multiform disorder of the bowels.

"From the species of flux (it has been already said) we could derive no criterion of the actual state of the disease, or its probable issue. Concomitant symptoms, which ordinarily afford an essential illustration of the nature of similar diseases, now rather contributed to perplex us. From the tongue nothing could be learnt; from the state of the abdomen very little; and, although pain and fever, and an excited pulse, necessarily implied a more active disease, they did not bespeak one more hazardous in the event. The danger was not to be estimated, either by the degree of pain or the degree of fever, or by the blood that was purged, or by the violence of the retching and vomiting, or by the frequency of the pulse. If any form of the disorder was more formidable than another, it was that which seemed to consist in mere diarrhœa.

"Two cases especially, which occurred soon after our first employment at the Penitentiary, made (as they were well calculated to do) a striking impression upon our minds. They had arrived, by a slow, and certain, and uninterrupted progress, at their fatal termination. The patients had no other symptoms of disease but a simple diarrhœa.

"They had no pain, no fever; their pulse was sixty, and no more. Never did I witness the process of dissolution so lingering. In the very act of dying, when the pulse could only just be felt, it had not exceeded sixty. Their purging was incessant and incontrollable; but there was no morbid quality in their evacuations, except that they were watery. One of these patients, a female, was examined after death, and the only traces of disease discovered were three or four small spots of ecchymosis at the upper portion of the large intestines, without any appearance of vascularity or inflammation in their neighbourhood.

"In process of time, cases of this kind became more and more numerous. They consisted of simple diarrhœa alone: such a diarrhœa as all physicians would have thought capable of being instantly arrested by the simplest remedies; yet this mere passive diarrhœa was the form of the disease which our own experience eventually taught us most to fear and deprecate.

"Many poor wretches thus affected were given up by us as lost, and were just ready to perish at the time when we first resorted to the use of mercury. They lay in bed without fever, without pain, without excitement of the pulse; but with a turbid water continually running from their bowels. This was their only symptom, and this nothing could restrain. Their complaint, as long as they did complain, was of 'that dreadful sinking.' But now their complaining had ceased. Being roused, they looked up for a moment, but made no lamentation, and then laid their heads down again in despair. It was a dismal office to watch over their tardy dissolution, and witness the frustration of every expedient for their relief. These were the cases in which we first put the efficacy of mercury to successful proof: and I cannot help mentioning the relief my mind experienced from a sense of responsibility which had now become truly awful, as soon as the salutary influence of this remedy was apparent. (P. 40—43.)

Dissection showed various morbid appearances, but none new or peculiar. There were spots of ecchymosis, but small (not exceeding the size of a split pea), and limited in number to five or six. They were found indifferently in all parts of the alimentary canal; and this was often the only morbid appearance in those who had died of "long-continued and uncontrollable diarrhœa." There were discolorations of the mucous membrane of every shade, from an indistinct blush to the deepest black: these assumed the appearance of distinct patches, each an inch or two in diameter, and terminating rather abruptly. They resembled spots of extravasated blood; but, on holding the part up to the light, it was ascertained that the blood was contained in vessels.

"The ulcers, which were found in the bowels of several who died of this disease, did not occupy especially those situations in which ulcers are most commonly met with,—namely, the termination of the small and the commencement of the large bowels,—but all situations indifferently. They were generally few in number, and occurred at distant intervals throughout the whole tract of the intestines. Sometimes (most frequently) they did not exceed in diameter the size of a pea, and were of a circular shape. Sometimes they were large and irregular, occupying in diameter a space of one or two inches. They did not appear in the midst of an inflamed surface, but were circumscribed and distinct; while the mucous membrane in their neighbourhood was of a perfectly natural colour.

"These ulcers had a certain correspondence with the ecchymosed spots and vascular patches already described. They were of the same size and shape, and, like them, they were found at intervals remote from each other, and without any marks of inflammatory action around them. These considerations led to the belief that what was now an ulcer had before been a spot of extravasated blood, or a patch of small blood-vessels in a state of congestion, and that one had terminated in the other. The ecchymosed spot, the vascular patch, and the ulcer, were found all three together in the same bodies; and the two first were sometimes found alone, and sometimes together. But the ulcer was in no instance observed without one or both of the other two. Indeed, the vascular patch was sometimes met with in (what appeared to be) its state of transition into an ulcer. In the space which it occupied, the mucous membrane was rough and unequal to the touch, and, upon close examination, appeared visibly corroded.

"The ulcers were discovered at various stages of their progress, in different bodies, and in the same body. Sometimes they had reached as far as the muscular coat of the intestines, and sometimes as far as the peritoneum, which alone remained to preserve the continuity of the bowel in these situations. In a few instances the perforation was complete; for they had penetrated the peritoneum, and reached the cavity of the abdomen.

"Generally there was a mere absorption of parts in the space which the ulcer occupied, constituting the simplest kind of ulceration. But occasionally there was something more than this: a foul yellow matter appeared in the centre of the ulcer. This matter was either the sphacelated structure of the bowel itself in the process of separation, or it was a morbid secretion proceeding from the ulcerated surface.

"Those who are conversant with morbid dissections, will certainly not discover any thing new in these states of ulceration. There was, however, one appearance, not unfrequently met with in our examinations, with which I was then unacquainted, and which, as far as I know, has never been particularly described. This was the appearance of ulcers in the course of their progress towards reparation. It is a question, I believe, whether ulcers of the bowels be capable of reparation at all. Here, however, there seemed to be sufficient evidence that they were so.

"In several bodies which we examined, one, or two, or three little spots were found, corresponding in shape and size with the smaller ulcers which have been noticed, where there was no remaining character of ulceration, but the mucous membrane apparently drawn and puckered, and its continuous smoothness interrupted. At these spots, closer examination, by help of a lens, discovered a circular margin, which was slightly elevated, enclosing a space which was slightly depressed. This space had a reticulated appearance, formed by minute white filaments of lymph, crossing each other in various directions, among which small red blood-vessels were visible. There was no unusual vascularity of the mucous membrane in the neighbourhood, nor any alteration of its natural colour; so that these little spots would probably have escaped our notice, had we not been habitually minute in our examinations. In a few instances, however, we were led to them by observing a peculiar condition of the peritoneal coat, which seemed here and there gathered up and drawn to a point, appearing externally as if a small portion of the intestine had been taken up by the forceps, and tied with a ligature on the inside. Wherever such was the condition of the peritoneum, upon examining the bowel within we found an ulcer in (what I presume to be) the course of reparation, exactly opposite to it. It is probable that, where such was the appearance of the bowel externally, the ulcer had originally extended to a considerable depth; that it had reached, perhaps, beyond the muscular coat, and that its reparation was in some sort necessarily effected at the expence of the peritoneum. Granulations springing from the bottom of the ulcer, as they contracted and coalesced, would pucker and draw together that part of the peritoneum from which they grew.

"Now it can hardly be doubted that the conditions which have just been described, really arose from ulcers in the course of reparation. They were found in the same bodies with ulcers in a progressive state, and with spots of ecchymosis, and patches of vascular repletion." (P. 47—52.)

We have no doubt whatever that Dr. Latham is correct in the explanation he has given of these appearances. We have seen ulcers in the bowels, along with spots which had every appearance of having been ulcers, which had assumed a healing process: we might almost say that some of them were cicatrised. It must be confessed that the morbid appearances described are by no means adequate to explain the phenomena which occurred; and we are perfectly satisfied that there preceded the more apparent symptoms, and existed simultaneously with them, some of those unknown changes of the constitution which, as the author observes, "will not bear to be spoken of with precision." Indeed, it appears that, so early as the autumn of 1822, the prisoners began to decline in health, although it was not till February 1823, that the more formidable epidemic broke out. We entertain no doubt that the causes of this visitation may be traced to confinement, mental depression, bodily labour, and starvation. This last expression may appear strong; but how can any milder term be applied, when we consider that one ox-head was made into soup for 100 men, or 120 women, being at the rate of an ounce and a quarter for each male prisoner, and of course still less for the females; and even this was but the soup, and not the solid meat. The rest of the diet was on the same liberal scale:—so much for practical economy. This system was adopted in July; and it was during the autumn of the same year that the sickness of the prisoners first became apparent,—viz. "they became pale and languid, and thin and feeble."

The method of treatment consisted, at first, in the administration of chalk mixture, with tincture of opium, aided by an improved diet; by which means the flux, which had affected between four and five hundred individuals, had almost disappeared by the beginning of April. At the end of the same month it returned; but now the same plan failed to afford any relief. Chalk mixture, opium, bleeding, blistering, astringents, aromatics, bitters, mucilaginous drinks, antimonials, ipecacuanha, glysters of starch and laudanum, sugar of lead, and infusion of ipecacuanha,—fomentations to the abdomen, stimulating liniments, flannel rollers, and the warm bath,—were all severally and conjointly employed; most of them without even temporary, and all of them without permanent relief. Indeed, the only remedy spoken of as affording any decided palliation was a kind of cataplasm, formed of—Empl. picis comp. ℥ijss. Empl. plumbi ℥ss. Opii duri contriti, Olei menthæ, Camphoræ āā ʒij. M. This was spread upon folds of linen, and laid upon the belly. It seems to have acted by keeping up a state of delirious insensibility to pain, manifested by "continual talking and laughter:" as soon, however, as the immediate impression made by the opium on the nervous system passed away, the symptoms recurred with unmitigated severity. Under these distressing circumstances, and after the failure of every other mean, recourse was at length had to mercury. Many considerations had hitherto prevented its exhibition, but principally the general denunciation of practical writers against this medicine in scurvy, or any disease allied to it. Now, however, the scurvy had almost disappeared, and a fair trial of mercury was resolved upon. The result is thus stated:—

"We first made trial of this remedy in those cases which our experience had brought us to regard with the greatest apprehension,—cases, if I may so say, of mere passive diarrhœa, where there was no excitement of the circulation, where there was little pain, and little of morbid quality in the evacuations; but where the evacuations were enormously frequent, and hitherto absolutely incontrollable. In these cases all medical expedients had failed, and we were now compelled to content ourselves with such temporary relief, and such short intervals of ease, as opium, administered in draughts or clysters, or in cataplasms, was able to procure. In our trial of mercury for these cases, we proceeded thus:—Equal quantities of hydrargyr. c. cretâ and pulv. ipec. comp. were made into pills; each pill consisted of five grains, (two grains and a half of each ingredient,) and one of them was administered three times a-day, to about twenty patients. Still there was no abatement of the diarrhœa. They were administered four times a-day, and still the diarrhœa continued. They were given five times a-day; when, upon our next visit to the Penitentiary, we found, among those who had taken mercury, one female in a profuse salivation, and the diarrhœa completely arrested in her, and in her alone. This poor creature had formerly had scorbutic spots upon the skin, at the same time that she suffered a flux of the bowels. The scorbutic spots had disappeared altogether; the flux had subsided, but returned; and that form of it which has been described had now brought her life into imminent hazard.

"In this instance, the salutary effect of mercury was unquestionable; and the condition of its success seemed to be, that it had procured salivation. We proceeded, therefore, more boldly in the use of it, still giving it in the same form, and in combination with Dover's powder. We increased the dose to those who already took it; and, as they became salivated in succession, they were all freed from the symptoms of their disorder. We subjected more and more of the prisoners to the same treatment, watching them carefully in the mean time, for the sake of still more confidently ascertaining the precise condition which was essential to the success of the remedy. This we uniformly found to be the production of salivation.

"Be it remembered that these cases, upon which the salutary effect of mercury was first proved, were those which occasioned us the greatest apprehension. Several of the patients were so feeble and emaciated, so pale and faded in their aspects, that while, on the one hand, we were feeling our way with the mildest preparation of mercury for the purpose of curing their disease, we were, on the other hand, administering wine and cordials for the purpose of upholding their existence.

"The success of mercury under these unpromising circumstances, led first to the more general, and finally to the universal, employment of it. We resorted to it in every case of flux, where the remedies hitherto used had not satisfied our expectation. In short, we resorted to it in every case without exception." (P. 65 – 68.)

We have already pointed out the similarity between the most severe form of diarrhœa as it prevailed at the Penitentiary, and that described by Dr. Baillie in the paper above alluded to: we have now to remark that Dr. B. points out mercurials as occasionally producing benefit, in the forms of hydrarg. cum cretâ, blue pill, and calomel; we are, therefore, somewhat astonished that this circumstance should not have directed the attention of the author and his colleague to this method of treatment at an earlier period. When once adopted, none can complain that it was not followed up with sufficient vigour; although the speedy recurrence of the disease, which almost uniformly took place, prevents us from joining in the high encomiums which the author has bestowed upon the value of the remedy.

"In several cases, in which the agony from tormina and tenesmus was extreme, and the evacuations were enormously frequent, and consisted altogether of morbid secretions or blood, fifteen grains of calomel and two grains of opium were given at a dose.

"The patients to whom so large a dose of calomel was given, were most attentively watched. Especial care was taken that nothing should divert it from its influence upon the constitution, and that every accidental inconvenience that might accompany its operation should be rendered as tolerable as possible. If the griping increased, they had peppermint water to have recourse to; if it still increased, they were to be largely and frequently fomented with flannels wrung out of warm water; and, if it still increased, they were to be supplied with small doses of laudanum at short intervals.

"It will hardly be expected that this single dose of calomel and opium could be effectual to the complete cure of the disease. The degree of relief which the patient experienced the next day, and the changes which his condition had undergone in the mean time, determined the manner of proceeding in the further treatment of the case.

"The dose of fifteen grains of calomel and two grains of opium, administered under such emergencies as have been described, had almost always the effect of calming the symptoms; but the degree of relief it procured was various.

"On the next day, we sometimes found that the patient had passed an easier night, that the evacuations had been somewhat less frequent, and the tormina and tenesmus had been somewhat moderated; but that, since the morning, the symptoms had become worse again, the pains were as severe as ever, and the evacuations as frequent, and quite unaltered in their appearance. Under these circumstances, fifteen grains of calomel and two of opium were given a second time.

"Sometimes, the day after the first large dose of calomel and opium, we found the relief which had been procured through the night still maintained, and the appearance of the evacuations changed in some obvious respect for the better. Perhaps they were now free from all admixture of blood, which they contained the day before. Under these circumstances, half the former dose of calomel and opium was given.

"Sometimes, the day after the first large dose of calomel and opium, we found the patient exulting that he had been cured as by a charm; that he had slept all night, and his pains were gone; and that he had had several evacuations, of which the two or three last were almost natural. With this sudden improvement, salivation had either already risen or it was at hand. Under these circumstances, the use of mercury was either suspended altogether, or small doses of calomel and opium were given until ptyalism appeared, which was generally obvious at our next visit.

"Our ultimate object, in all cases, was to produce salivation. But, in these cases of severer suffering, we found a salutary impression capable of being immediately produced by a few large doses, or even by one large dose of calomel and opium. This it was expedient to make the most of. Nevertheless, this immediate salutary impression was soon lost, unless the same practice was followed up to salivation; for which purpose mercury was afterwards sparingly or largely exhibited, according to the circumstances which have been set forth.

"It was remarkable that the constitutions of many were most slow and reluctant in admitting the peculiar influence of mercury, and that the disease, in the mean time, was still unyielding. In such constitutions, after calomel and opium, given internally, had failed of their accustomed effect, and now served to irritate rather than to soothe the bowels, we resorted to inunction; and salivation, thus procured, was productive of the same benefits.

"As an object of pathological interest, it was instructive to observe the different modes of curative action by which, under the influence of mercury, different constitutions set themselves free from their disease. In many, the process of cure was so gradual, that there was hardly any perceptible action engaged in it. The constitution seemed rather to lose the disease, one symptom after another, than to surmount it by an effort of its own. The pains became gradually less and less, until no pains whatever remained; and the healthy secretions gradually predominated over the morbid, until all were healthy.

"In many the process of cure was by a sudden, vigorous, and painful effort, when the constitution threw off its disease by a sort of critical paroxysm. Sometimes the critical effort commenced as soon as the mercurial fœtor was perceptible in the mouth. Sometimes salivation would exist twenty-four hours before the crisis began; and sometimes the crisis preceded the salivation twenty-four hours. But it never took place but where there was salivation at the time, or immediately before or immediately after." (P. 69—74.)

The disorders of the *brain and nervous system* were various and peculiar, consisting of head-ache, vertigo, and apoplexy, of cramps of the limbs and convulsions, and, in some instances, of a sudden and excruciating agony, referred to the region of the heart, with death-like fainting. There were likewise several cases of phrenitis; but even here the disease did not assume its usual character, so as to render the common remedies available. The disease of the bowels, and these various affections of the nervous system, were sometimes observed separately, but more generally (especially after a term) they became co-existent in the same individuals. One of the most usual among the varied train of symptoms was as follows:—The patient was attacked with head-ache, while the bowels yet remained free from disease; under which circumstances, some of the patients were merely watched, without any remedies being administered; while others were treated with such means as would have been adopted in common practice. In almost every one, some form of the abdominal disease supervened. Even the more sudden and violent nervous affections were invariably followed, in a few days, by the flux, in some of its varieties. In other instances, the nervous symptoms supervened in the course of the bowel complaint. In short, the diseases, or rather symptoms, were so blended and associated, as to show unequivocally the identity of their origin.

The post-mortem examinations were necessarily performed under unfavourable circumstances, owing to the delay which occurred before the verdict of a coroner's jury had been returned, prior to which no dissection was permitted. Some vascular fulness of the brain, and its membranes, is mentioned, together with effusion of small quantities of fluid between the membranes, or into the ventricles. But from this, little is to be learnt.

The author seems to regard some explanation as necessary to justify the treatment adopted: it is fairly and judiciously given as follows.

"There are ailments concerning which physicians hardly allow themselves to feel the smallest anxiety, holding (as they conceive) in their own hands the almost certain means of their relief. When, however, such ailments unexpectedly resist the remedies which are accustomed to cure them, they cannot help suspecting that there is more to contend with than the mere symptoms seem to imply. Thus, simple pain in the head they are apt to think lightly of, until it is found incapable of relief by common remedies. Of such intractable cases, the number, it has been seen, was very large at the Penitentiary.

"Of pain in the head accompanied by vertigo, they are apt to think a little more seriously, but still not very seriously, until simple remedies bring no relief; and intractable cases of this kind, also, it has been seen, were very frequent at the Penitentiary.

"Again, there are many, even acute diseases of vital organs, which physicians always regard with great apprehension, but which, with the advantage of seeing and treating them early, they nevertheless have a good expectation of bringing to a favourable termination. When, however, even with this advantage, they absolutely fail in every case that presents itself, they cannot help experiencing some perplexity, and suspecting that there may be more in these diseases than they have been able to discover. Thus, when to pain in the head, or vertigo, there are superadded cramps of the muscles, or twitching of the tendons, or delirium, or strabismus, they look upon the cases constituted of these symptoms with the greatest apprehension of the result; and there were, it has been seen, a few such cases at the Penitentiary. But, even in the cases constituted of these symptoms, they do not look upon death as inevitable, until the remedies, upon which they are accustomed to rely, fail to make their ordinary impression. And thus it happened with the few cases of this kind at the Penitentiary: they passed uninterruptedly to their fatal termination, their symptoms hardly receiving the smallest check or abatement from the remedies employed.

"Now, before we resorted to the use of mercury for the various forms of the disease prevalent at the Penitentiary, the state of the prison, in regard to that form of it which involved the brain and nervous system, was this:—Seven had already perished under our own observation; of whom, one died apoplectic, one maniacal, two with the symptoms of phrenitis, two from cramps referable to the region of the stomach and the heart, and one from symptoms belonging in part to the heart and in part to the brain; and there were not less than two hundred now labouring under various degrees of disorder belonging to the same organs. Of them a few only were dangerously ill, in respect of the magnitude of their symptoms. These few were suffering that insidious form of phrenitis already described, while we were checking the symptoms, and postponing the progress of their disease, with little hope of eventually saving their lives; and, unquestionably, they were in great present peril. But all the rest, though not in imminent danger from the magnitude of their symptoms, gave just cause for anxiety, from the consideration that they had not been cured by any means hitherto employed; and, moreover, that they were under the same conditions of disease through which the seven had passed, before they reached their fatal consummation." (P. 103—107.)

It is sufficient to observe, that mercury was had recourse to, almost as an universal remedy. Instead, however, of entering into any general description, we shall content ourselves with one case in illustration.

"One of these cases was peculiarly striking, and is well worth relating as an example. Upon our first visit to the Penitentiary, on the 1st of March, we found, among the patients in the infirmaries, a young man of the name of Robson. He was twenty-one years of age; and told us that he had suffered a head-ach of the most excruciating kind during several months. His sight was dim, and he had a constant twinkling of the eyelids, and great agony was depicted in his countenance. He had, moreover, that faded, pale, and melancholy aspect already alluded to. Yet the functions of his bowels were performed naturally; his tongue was clean; and his pulse was of the natural frequency and strength. At this time the alliance between the disorders of the nervous system and the bowels was hardly ascertained; therefore, no inquiry was made whether he had suffered diarrhœa. Subsequently, however, we learnt that, during the preceding winter, his bowels had been twice slightly disordered for a few days. This poor fellow was under our constant observation and treatment, from the beginning of March to the end of June. He gained no respite from his agony, but by means of leeches applied to the forehead, day after day, for a week together, or by blisters, kept open, or applied in quick succession, behind the ears, or on the nape of the neck; and the respite thus obtained was of short duration: it might be for ten days; it was never longer than a fortnight; and then the same agony returned, and the same cruel treatment was to be resumed. In this case we eagerly resorted to the use of mercury: salivation was procured, and maintained to a certain degree, during several weeks. Whereupon the patient was released from his misery, and ever afterwards, during eleven months that I had the opportunity of watching him, he continued free from complaint of every kind." (P. 109, 110.)

When the disease was comparatively mild and slow in its progress, salivation was procured *gradually:* when the disease was more severe and rapid, it was effected as speedily as possible. Thus, to some, one, or perhaps two, grains of calomel, with a little opium, were given once or twice a-day; to others, ten, or even twenty, grains of calomel, with a comparative quantity of opium, once or twice in twenty-four hours.

*The Fever* is regarded by the author as having been *sui generis*, and idiopathic. Sometimes it existed alone, and at others was co-existent with the other forms of the disease. It generally disappeared altogether within a week, when it was mild; and, whether mild or severe, if continued beyond this period, the type changed, and the fever became hectic.

This chapter is far from being devoid of interest, and we regret that our narrowing limits oblige us to give a less full account of it than of those which have preceded. The cases detailed, particularly that of Mary Chapman, are highly important: but we must content ourselves with these general expressions, and conclude by stating that the treatment which was found most efficacious consisted in free evacuations during the early stages, and the administration of bark and acid as soon as the internal feelings of oppression had subsided, leaving only the fever and symptomatic sweats. Thus continues our author:—

"Thus I have described the complaints prevalent at the General Penitentiary, as they fell under my own observation. These were a scurvy, a flux of the bowels, a disorder of the brain and nervous system, and a fever. All four, from their extent and frequency, had a claim to be considered *the disorders of the prison;* and the manner in which they were often combined in the same individuals, and the way in which they were taken up and succeeded by each other, induced the strongest belief that they all sprang from the same cause, and were, in a certain sense, the same disease. This belief was further confirmed, in respect to two of them, the flux of the bowels and the disorder of the brain and the nervous system, by the consideration that they were both amenable to one and the same remedy, and (as far as our experience went) intractable by any other.

"What then, it may be asked, was the essence of the disease, considered as a whole? Of the inceptive error, or primary morbid action, from which it arose, I am entirely ignorant: whether it belonged to the blood-vessels or the nerves, or to what particular organ or system of organs. I only know that its first cognizable effects were seated in the blood-vessels, and that they consisted both in the admission of blood into their capillaries, beyond the natural sphere of the circulation, and in its transmission through their capillaries, out of the sphere of the circulation altogether;—that the consequences were ecchymosis of the skin, and ecchymosis and vascular patches of the mucous membrane of the stomach and intestines, and determinations of blood to the brain and its membranes, and (probably) to the spinal marrow;—and that out of these arose diseases, which obtained different names, according to the manner of their occurrence and the parts upon which they fell, —such as the scurvy, and the various species of flux, and the various species of nervous disorder, and some forms of fever; all of which were, nevertheless, still the same disease in the conditions of their production.

"I am well aware that the essence of the disease must have consisted in something prior to these effects, and productive of them. But the present state of our knowledge does not enable us to ascertain what it was: and, indeed, the search after the essence of any disease, beyond the point at which it begins to fall within the reach of the senses, has seldom brought the pathologist to any more certain conclusion than this,—viz. that it consists in 'a morbid disposition, or action, which is *sui generis.*'" (P. 137—140.)

*The Intercurrent Diseases* were some cases of erysipelas, of swelling and inflammation of the labia pudendi, terminating in abscess,—accidental sores,—and extensive ecchymosis, occasionally following the application of leeches. Of all these it is proper to state, that they were more or less modified by the prevailing epidemic; but there is nothing else which appears to us worthy of being more particularly specified.

Having closely followed our author thus far, we shall discuss very briefly what remains,—viz. the extent to which the epidemic prevailed, and the effect of removing the prisoners from the Penitentiary.

The extent of the evil will appear from the following tables; in which, by *ill* is to be understood those in whom the disease was either stationary or progressively getting worse; by *better*, who had made some advance towards health; and by *well*, who were free from symptoms of the disease, but deemed prone to relapse.

"Prisoners under Medical Treatment, on the 15th of May, 1823:—

| | | Men. | | Women. | | Total. |
|---|---|---|---|---|---|---|
| Diarrhœa | Ill . . . | 46 | — | 44 | — | 90 |
| | Better . | 48 | — | 87 | — | 135 |
| | Well . . | 49 | — | 20 | — | 69 |
| Other Complaints . . | | 21 | — | 30 | — | 51 |
| | Total . . . | 164 | — | 181 | — | 345 |

"On the 23d of May:—

| | | Men. | | Women. | | Total. |
|---|---|---|---|---|---|---|
| Diarrhœa | Ill . . . | 63 | — | 46 | — | 109 |
| | Better . | 51 | — | 56 | — | 107 |
| | Well . . | 64 | — | 47 | — | 111 |
| Other Complaints . . | | 24 | — | 36 | — | 59 |
| Total . . . | | 202 | — | 184 | — | 386 |

"On the 11th of June:—

| | | | | | | |
|---|---|---|---|---|---|---|
| Diarrhœa | Ill . . . | 73 | — | 35 | — | 108 |
| | Better . | 86 | — | 38 | — | 124 |
| | Well . . | 82 | — | 88 | — | 170 |
| Other Complaints . . | | 24 | — | 28 | — | 52 |
| Total . . . | | 265 | — | 185 | — | 454 |

"On the 3d of July:—

| | | | | | | |
|---|---|---|---|---|---|---|
| Diarrhœa | Ill . . . | 17 | — | 22 | — | 39 |
| | Better . | 70 | — | 37 | — | 107 |
| | Well . . | 179 | — | 83 | — | 262 |
| Other Complaints . . | | 8 | — | 22 | — | 30 |
| Total . . . | | 274 | — | 164 | — | 438." |

Thirty died under the care of Dr. Latham and Dr. Roget; of whom, however, only twenty-two were carried off by *the disease*.

Notwithstanding the effects of mercury in arresting the various forms of this epidemic, relapses soon began to occur, and at length became very frequent; a circumstance which naturally tended to increase the anxiety attending a trust of such vexatious responsibility, and induced the medical attendants to request the assistance and authority of some of their professional brethren. In consequence of this requisition, they were joined by Drs. Hue, Macmichael, and Southey.

The sickness still continuing, although in an abated degree, and numerous relapses taking place, at first a part, and ultimately the whole, of the prisoners were removed. When the experiment was first tried, it was attended with the happiest results, especially to the men who were sent on-board a hulk at Woolwich. Notwithstanding these favourable prospects, the tendency to relapse could not be overcome; some having a recurrence of the disease after intervals of three, four, and five months, and after having apparently regained a state of robust health. To conclude, it was found necessary to discharge all the women, the severity of their sufferings being taken in lieu of a more protracted confinement; and the men were distributed among the hulks. While they remained Penitentiary prisoners, they could not be subjected to hulk discipline; but the power of transferring them having been procured by an especial Act of Parliament, they were now taken on shore to work; and the accounts from the different stations to which they were sent, seem to show that the disease has at length been fully overcome.

There still, however, remains a very important part of the question to be considered: we mean the origin of the disease. At first this was attributed to the impoverished nature of the diet, and the severity of the winter; and these were assigned, in the first Report, as the *exclusive* causes. But, when the disease returned after these causes had ceased to operate, and particularly when it was found intractable by ordinary means, the physicians were driven to the necessity of seeking some other explanation; and accordingly, in their Report, dated July 3, 1823, they intimated to the Committee that they suspected the operation of other causes; hinting at contagion as one of these, and confidently asserting the existence of an "injurious influence peculiar to the place itself."

We have perused with care all the arguments,—or, rather, the relation of all the circumstances which might be regarded as arguments, in favour of the former hypothesis; but, we confess, without being by any means satisfied of any contagion having existed. When a number of individuals are exposed to other causes, it appears unnecessary to seek for an explanation of such partial application as contagion would afford, even if its existence was clearly made out. That many had the disease altogether independent of any contagious influence, is unquestionable: that any had it from this cause, appears (to say the least of it) very problematical. Indeed, the only case affording any thing like a direct instance of contagion, is that of a waterman, employed in communicating between the Narcissus hulk and the shore: he had a severe attack of dysentery. Many such cases, had they occurred, would have afforded strong presumptive evidence; but a solitary instance proves nothing. It is proper to state that Dr. Latham does not attach much importance to it himself, and is evidently very doubtful as to the truth of contagion having existed; although this opinion is warmly advocated by Mr. Holford.

The question of a noxious influence in the place itself is next discussed at great length, and stands upon very different grounds from the preceding. It is unquestionable that confinement, mental depression, and impoverished diet, must have had a great share in the production of this epidemic; but as all these, excepting the last, must be supposed to exist to an equal extent in other prisons, where no such disease has prevailed, it is obvious that some other cause, not in general operation, is required to explain the effect. Dr. Latham and his colleagues regard this as consisting in a "noxious influence" endemic in the place.

It appears, from the books of the apothecary, that very large chalk instead of mutton quantities of chalk mixture had been served out to the prisoners ever since 1816; and this gentleman, on being questioned by the physicians, stated the disease for which he prescribed it to have been diarrhœa. Upon these grounds, apparently very strong, it was that Dr. Latham and his colleagues drew up a formal report, stating it to be their opinion that diarrhœa had been endemic in the Penitentiary ever since its establishment, and had only been aggravated by circumstances into the malignity it recently displayed. This is a very important question, as it involves the expediency of continuing or discontinuing to use the Penitentiary as a place of confinement; and, therefore, requires deliberate investigation.

In the first place, we have the apothecary's books to show that quarts and gallons of chalk mixture have been habitually given to persons under his care; and for what complaint but diarrhœa, asks our author, could these medicines have been administered? In the second place, we have the direct testimony of the said apothecary, who, being asked "Do you give the chalk mixture for any other disorder but diarrhœa?" answers "No." Now all this appears very satisfactory and conclusive; and, although it might be matter of astonishment that Dr. Latham should bring forward the evidence of the apothecary, in preference to that of the medical superintendant, yet so long as his evidence remained unimpeached, it could not affect the accuracy of the conclusions drawn from it. But the great error (as it appears to us) of Dr. Latham and his colleagues consisted in founding their opinions on the authority of Mr. Pratt's evidence; a foundation utterly unfit to support any superstructure whatever.

We have seen above that our author quotes the authority of Mr. Pratt, to prove that he never gave the chalk mixture for any thing but diarrhœa; yet, in another part of his evidence, which Dr. L. does not quote, he expressly states that he gave it to every prisoner who complained of a pain in his bowels; while in yet another part he acknowledges the greater number of his patients to have been impostors.* And when we consider that the mixture in question contained *half an ounce of tincture of calumba, two drachms of aromatic confection, and twenty-five drops of laudanum*, we cannot be surprised that it should be in great request among persons who had been accustomed to live freely, and who were now denied every other means of obtaining any thing which could remind them of their former pleasurable sensations. If, from the confused and contradictory testimony of the Apothecary, we turn to the evidence of the Superintendant, we find this to be the true explanation of the extraordinary demand for Mr. Pratt's chalk mixture.

* See Printed Evidence, p. 43; and Mr. Holford's "Third Vindication," p. 63 and 86.

"Q. Were you not in the habit of going round the prison at the beginning of every month, with Mr. Pratt, accompanied by the governor, in the part of the prison occupied by males, and by the matron in the part occupied by females, and examining every prisoner personally as to the state of his health?—A. Yes, I was.

"Q. And were not most of the Monthly Reports signed by yourself, as well as Mr. Pratt?—A. They were, undoubtedly, till June 1822; when, according to my instructions, the Quarterly Reports only were signed by myself.

"Q. Is it possible that a diarrhœa of a peculiar character, difficult of cure, could have escaped your observation during that time?—A. *It is impossible*."—(*Printed Evidence*, p. 52, 53.)

Neither are we satisfied with the manner in which the calculations respecting the number of patients supposed to have laboured under diarrhœa were made. The following is Dr. Latham's evidence upon this point:—

"Q. Were Mr. Pratt's accounts so clearly kept, that the name of every person who had taken physic always appeared?—A. We could only reckon upon what did appear.

"Q. For instance, if Mr. Pratt's entry is 'Mrs. Clarke's women, a mixture,' in what way is that entered in the tables?—A. I perfectly recollect that some abatement was made, but of what kind it was, I cannot state at this moment: but I know that some abatement was made upon that consideration.

"Q. For instance, there is 'the kitchen women, a mixture;' 'Mrs. Clarke's women, a mixture;' and 'Mrs. Todd's women, a mixture:' in what way, in forming the tables, were these entries used?—A. I know that some abatement was made, which we considered to be a just abatement; but of what kind it was I forget.

"Q. Are you aware that Mrs. Clarke's women might be as many as thirty?—A. Yes.

"Q. In what way was that noticed in the table?—A. I quite forget; I am not even certain whether we did not exclude the whole of that."

Mr. Pratt's testimony goes to prove still more strongly that these calculations were merely conjectural; but the argument must be very weak which requires the assistance of his evidence. It likewise appears that this lavish expenditure of chalk mixture was greatly diminished from the time that Mr. Hutchison was requested "to sign, as approving thereof, all demands for medicine, &c." Hear Mr. Holford:—

"To the appointment of Dr. Hutchison with these instructions, rather than to any change in the health of the prisoners, I attribute the decrease in the comparative numbers of those who were (in the language of the physicians) treated for diarrhœa; or who (to speak in plain terms) took one or more doses of chalk mixture, or chalk powder, during the latter years mentioned in the tables of the physicians. It was not very likely that a physician of long practice in the navy and in hospitals, like Dr. Hutchison, when it became a part of his duty to examine and check the expenditure of the medicine used in the Penitentiary, should allow Mr. Pratt to go on sluicing out his chalk mixture from his stores in quarts and gallons, and white-washing, in this manner, the intestines of a whole ward at a time. I really recollect nothing like this in modern practice, except in the case of a physician in a farce, (one of Foote's, I believe,) who, having on one day ordered the right ward to be purged and the left to be bled, prescribed on the next day the bleeding for the right ward, and the purgatives for the left." (P. 36, 37.)

But, further, with regard to the chalk mixture, it would seem that, where simply complaining of a pain in the belly was not sufficient to procure a dose or two from the bountiful hand of Mr. Pratt, *diarrhœa was feigned for that purpose.* Dr. Latham does not believe this; but the testimony of the Superintendant is too strong to be easily set aside.—"I have also known convicts in the Penitentiary at Millbank, previous to the late malady breaking out, both in their cells and in the infirmary, break down, with their fingers, in their urinary utensils, a good figured or formed motion, and intimately mix it with the urine, so as to induce the belief that it was in reality a diarrhœal evacuation; but a little attention to the character and appearances, with strict watching, will in most cases lead to a detection of the imposture. The object which convicts have in such practices, is to be exempted from labour, and to be kept in the infirmary, where their comforts in every way are certainly much greater than when in their cells in the prison."* Nay, Mr. Hutchison has even stated, that he has known the prisoners in the Penitentiary barter their stools to some of their companions, who were desirous of feigning a very copious flux.

The question stands thus:—Is the circumstance of a large quantity of chalk mixture having been consumed in the Penitentiary, sufficient ground for believing that diarrhœa has always prevailed there; and, consequently, that the place is unhealthy? We think not,—because the circumstance may be explained (and is sufficiently explained in the evidence we have given above,) on other principles; because the testimony of the more credible of the two medical officers is directly opposed to this supposition; because the testimony of the other, on which the physicians have founded their opinion, is contradictory, and therefore nugatory; because diarrhœa does not prevail in the dwellings situated in the neighbourhood, nor in the regimental hospital of the Grenadier Guards,† in the same locality and immediate vicinity; because the severity of the season, and reduction of diet, appear to us sufficient to explain the phenomenon; and, to conclude, because, these last-mentioned causes

* Mr. Copland Hutchison on Simulated Diseases, in the *London Med. and Phys. Journal*, February 1824.

† Both the Editors were formerly on the medical staff of this regiment, and can bear testimony to the accuracy of this statement.—Editors.

being removed, no diarrhœa prevails. "Perhaps," says Mr. Holford, "the best answer that can be made to those who are inclined to suspect the existence of some influence injurious to health in the site of the Millbank Penitentiary, is, that the prisoners who now inhabit it are healthy; many of them being much better in health than they were when they came there." —(*Third Vindication, &c.* p. 1.)

---

ART. III.—*An Essay on Venereal Diseases, and the Uses and Abuses of Mercury in their Treatment. Illustrated by Drawings of the different Forms of Venereal Eruptions.* Second Edition. By RICHARD CARMICHAEL, M.R.I.A. Vice-President of the Royal College of Surgeons in Ireland, and one of the Surgeons of the Richmond Surgical Hospital, Dublin, &c. &c. &c.—8vo. pp. 376. Longman and Co. London, 1825.

[Concluded from page 417.]

HAVING, in our last Number, given rather an extended account of the first chapters of Mr. CARMICHAEL's work, and concluded our analysis of his first class of venereal diseases (the *papular)*, we resume our task at page 148, which brings us to the fourth chapter, treating of the *Pustular Venereal Disease.* We copy the description of the primary ulcer, which Mr. C. describes as the parent of this class of constitutional symptoms.

"The primary ulcer of this disease is characterised by a reddish-brown surface, which borders closely on the phagedenic character. Its edges are raised, and well defined. It is not excavated, but is either on a level with the surrounding skin, or considerably raised above it. At its commencement it appears in the form of a small pustule, attended with itchiness of the part.

"It may be distinguished from the primary ulcer of the papular disease, by its well-defined and elevated edges, and also by the absence of the smooth fungous appearance which characterises the second stage of that ulcer. From the phagedenic primary ulcer, to be described in the next chapter, it may be distinguished by its well-defined margin, and from the absence of the irregular and corroded-like surface of that ulcer, and also by its exemption from the painful and acute symptoms by which the phagedenic ulcer is characterised. It may also be distinguished from chancre, by its want of the callous edge and base which always attend that ulcer. The ulcers of which we are treating are most frequently found on the external surface of the prepuce, body of the penis, and occasionally on the scrotum, and occur from the size of the smallest pea to that of a shilling. Of the former size, they frequently form a circle round the orifice of the prepuce, and occasion, when the ulcers heal, a permanent phymosis, which can only be removed by the knife. They are, in general, of a chronic nature, and evince but little disposition to spread.

"Mr. Evans conceives that this primary ulcer, and that of the papular disease, are one and the same; and that I have been led into the error of supposing they were essentially different, by having observed the same ulcer in different stages of its progress, when it exhibits different characters. He supposes that this ulcer, which I have said has 'the appearance of excavation,' is nothing more than the primary ulcer of the papular disease in its ulcerative stage, which, in general, ceases about the eighth day, when the ulcer begins to assume its raised, smooth, fungous character. In confirmation, however, of my own opinion, I have only to observe, that the primary ulcers I am considering will exhibit for weeks, nay sometimes months, the same excavated appearance, arising from the same raised edges which they displayed in the first week after their attack; and that they do not, during their entire progress, exhibit the smooth fungous surface of the primary ulcer of the papular disease.

"Besides, in those cases in which I have been able to trace this ulcer to its constitutional symptoms, they were essentially different, as I shall presently show, from those which attend the simple primary ulcer, considered in the last chapter; and these circumstances, I conceive, sufficiently warrant me in the assumption that these ulcers are essentially different, and arise from different poisons. I must, however, admit that I have not met with many instances in which I was enabled to follow this ulcer to its constitutional symptoms.

"In the Appendix to my former edition, I detailed two cases, in both of which the eruption was pustular, and afterwards spread into ulcers, covered, on their first formation, with *thin* crusts.

"In the London Medical Journal for 1815, I have given a considerable number of examples of this species of ulcer, three of which were attended by constitutional symptoms. The eruption in each was preceded by fever, and consisted of phlyzacious pustules, some of which declined, while others ulcerated, and were covered with thin scaly crusts." (P. 148—150.)

Our author further observes, that the buboes which arise from this virus resemble the primary ulcer, inasmuch as they have a tendency to form undermined or projecting edges, especially where much mercury has been employed; and he observes, that caustic is so slow in its operation upon these edges, that he always prefers removing them with the scalpel. In the treatment of this kind of primary ulcers, Mr. Carmichael objects to the use of mercury, as it often fails in inducing them to heal, and they appear to be particularly obstinate under its use: neither do stimulating nor caustic applications to the part produce any better effect. Absolute rest, antimonials, and sarsaparilla, form the *methodus medendi.*

Several cases, illustrating this species of ulcer, occupy the greater portion of this short chapter. In the three first cases, the ulcers had existed five or six weeks, and no mercury had been used. These cases are, on many other accounts, well worthy of attentive consideration. These details are followed by some remarks upon the tendency of all eruptions, whether venereal or not, to glide into those of the nearest character. Thus, chicken-pox, which is a vesicular disease, will often be mixed up with pustules, closely resembling those of small-pox, and *vice versa.* Itch, our author also remarks, is to be found exhibiting pustules, vesicles, or papulæ, all at the same time. So likewise the venereal eruptions are liable to the same anomalies: for example, the papular eruption may exhibit a few pustules, which, like the pustular venereal eruption, form thin crusts, instead of ending in desquamation; and the pustular disease may exhibit papulæ among the pustules. But this practical truth, he continues, is worth adding—that the more closely the eruption approaches to the form of papulæ, terminating in desquamation, so much the more mild, yielding, and manageable will the disorder be found.

We particularly call the reader's attention to the above paragraph, because we see in it a virtual abandonment of all the refinements of our author's theory, and an easy escape from any dilemma which the mixture of papulæ, pustules, or scales, may produce; and which may enable the ingenious practitioner to reconcile the rebellious eruption to that species of sore from which it *ought* to have its origin.

Our author, in concluding this chapter, remarks that the pustular venereal disease, both primary and secondary, forms the natural link between the papular and phagedenic diseases; but he defers treating of the constitutional symptoms, until he shall have described the phagedenic ulcer; and this brings us to the longest and best-written chapter in the volume.

*On the Phagedenic Venereal Disease.*—Our author, in this division of his work, describes two kinds of sores,—the phagedenic and the sloughing. The first, he says, has a corroded appearance, and does not exhibit granulations nor surrounding induration: sometimes it spreads rapidly, at others more slowly; but, instead of being checked by mercury, it is rendered more inveterate and rapid in its progress by that mineral. Its most frequent seat is the glans penis, and it may proceed to the total destruction of the part; but a spontaneous hemorrhage will occasionally occur, and check its course. Another characteristic circumstance is the frequent return of ulceration, after the part has healed, to the very spot first affected. Mr. C. observes, in continuation, that he never yet had an opportunity of seeing this ulcer in its commencement, and is therefore ignorant whether it arises from a pimple, pustule, or vesicle, or whether it passes immediately from its commencement into its phagedenic state. And the same remark applies also to the sloughing ulcer, which is still more untractable and destructive than the one just described. A small black spot first denotes the commencement of the sloughing process, which will increase occasionally to three or four times its original extent, and then separating, does not leave a clear surface beneath, but a corroding sore, which begins a new depredation on the parts beneath. Violent pain attends this form of ulceration; and the ravages it is capable of committing upon the neighbouring parts may readily be imagined by the experienced surgeon.

Our author further observes, that these sores may readily be distinguished, after they have assumed their peculiar characters, from all others; but, as he is entirely ignorant of any criterion by which they may be known in their infancy, is he not asking too much when he wishes us to believe that they are the results of a specific poison? especially when he admits that excessive constitutional irritation will cause a venereal ulcer, as it will any other, to become phagedenic, however mild in its nature. Well he may say that this admission "may seem to favour the opinion of his adversaries," (that is, we presume, the adversaries of his theory; for we would wish a strong line of distinction to be drawn between the opponent of *the doctrine* and the opponent of the *man;)* for, in fact, it is conceding the whole question: and though we shall have occasion, in the review of this chapter, to express our approbation of our author's accurate description and sound practice in this class of complaints, we must again take occasion to lament that he should have thought it necessary to dwell so long upon, and to strain so hard to maintain, doctrines that are too refined for common observation, and tend rather to confuse than elucidate his subject. There can be no doubt of the truth of his observation, that mercury, improperly exhibited, will produce irritation and inflammation in a simple ulcer, as will also neglect and imprudence of various kinds; and this inflammation may go on to produce mortification: but then, he adds, when the slough separates, a clear granulating sore is exposed; whereas, in the case of the sloughy sore, a phagedenic surface presents itself upon the slough falling off.

Buboes are more unfrequent in the case of the sloughy and phagedenic ulcers, than in any other; and this is what might reasonably have been expected, from the rapidity with which the parts are destroyed. To this cause we must recollect that an eminent authority on this subject long since attributed the infrequency of constitutional symptoms following this particular sore.

We next come to the consideration of the constitutional symptoms derived from the phagedenic and sloughy sore; but, before we begin to describe them, we must observe that we do not think Mr. Carmichael has dwelt with sufficient force upon the great constitutional disturbance produced by the sloughy chancre. The pain is usually very severe, the fever considerable, the thirst intense, and, in short, the whole system so disordered, as to preclude, we should hope, the most decided mercurialist from any attempt to administer that remedy, where it is so strongly contra-indicated; though there is a sore which frequently is found in the angle between the internal prepuce and glans,—which spreads by sloughing rather than by ulceration,—which is surrounded by marked and extreme induration, but unattended with pain or fever, in which the internal exhibition of mercury produces the most beneficial change; and the local action of a mercurial wash upon the part is equally efficacious. Very numerous examples of this form of ulceration have come within the scope of our observation; and we are, therefore, somewhat surprised that Mr. Carmichael has said nothing of it.

Returning, however, to our author's work, we find the constitutional symptoms of the phagedenic and sloughing ulcers thus described:—1st. An eruption of tubercles, pustules, or spots of a *pustular tendency*, which quickly degenerate into ulcers covered with thick crusts, usually healing from the centre, at the same time that they are extending at their circumference with a phagedenic border. The eruption is often ushered in by a high degree of fever; at others, by a lesser degree of general ailment. In other instances, severe nocturnal head-aches, with tenderness of the scalp, dyspnœa, with tenderness of the sternum, and general soreness of the chest, occur. These, however, our author admits not to be peculiar to, but only more severe in, this form of disease. 2d. The ulceration of the throat attending this disease, is of a very formidable nature, beginning with a small apthous-looking spot about the velum, or posterior part of the pharynx, extending up the nares, frequently followed by caries, with exfoliation of the spongy bones, and discharge from the nostrils. If it should extend downwards to the larynx, as is not unfrequently the case, there will be but little chance of saving the patient's life. Our author affirms that he has never found this affection of the nose connected with the papular eruption, or the scaly syphilitic lepra, or psoriasis; but in every instance the eruption was pustular, and had either formed scabs or ulcers. This is a point in which argument is of no avail; but we have no hesitation in distinctly denying this assertion, and could produce, from our own experience, decided cases of an exactly opposite tendency; and we fearlessly appeal to the testimony of practitioners in general for the truth of our assertion. 3d. Pain in the knees, wrists, and ankles, which often become swelled, red, and painful. The affection of the knee is, says our author, a frequent occurrence, so much so as to be esteemed one of its peculiar characters; and is attended by all the symptoms denoting an acute attack of the synovial membrane.

On the subject of nodes occurring in this form of the disease, Mr. C. is not quite positive; though he has never seen an instance of them where mercury had not been previously employed. Many anomalous symptoms are occasionally met with, among which an enlargement of the testes may be mentioned. Of others, it appears probable that the injudicious employment of mercury may have given rise to them.

The cure of the phagedenic ulcer is to be effected by rest, venesection, antimonials, warm poultices and warm fomentations, opium, hyoscyamus, or cicuta, to lessen pain and procure rest at night. When the inflammatory stage is overcome, to check the phagedena, solutions of lunar caustic may be employed, or the black or yellow mercurial washes; or, where all these means fail, paring off the edges of the sore, and encouraging the hemorrhage by immersing the parts in warm water, has been found successful. Stimulating applications, too, are often of service in these ulcerations,—such as turpentine or balsam of copaiba. In this stage of ulceration, our author disapproves of emollient poultices: nor has he a better opinion of the internal exhibition of bark, which has been recommended.

We regret exceedingly that it is totally impossible for us to extract any portion of our author's very interesting cases; and, consequently, we must refrain from any comment upon them. We can do no more than refer our readers to the original work.

On the subject of the treatment of the constitutional symptoms, we find that, if the eruption is ushered in by much fever, and the pains in the head and joints are severe, blood-letting may be necessary: opening medicines and antimonials, with low diet and confinement to the room, constitute the chief means of cure during this stage. But, if the fever be inconsiderable, the decoction of sarsaparilla, combined with antimonials, will effect the cure. If constitutional ulcers exist, cicuta, in full doses, may be advantageously combined with the sarsaparilla. In those cases in which tubercles are formed, followed by foul and extensive ulcers, Mr. C. believes the nitrous acid to be particularly useful. Many auxiliary remedies are mentioned by our author, to mitigate particular symptoms, but the above forms the essential principles of his method of treatment; and it is only on the subsidence of the disease, when the fever is gone, the constitutional ulcers are healed, and the most recently occurring pustular or tubercular spots have terminated in a kind of *scabby desquamation*, that mercury may be employed, as it will then hasten the cure, and does not risk the chance of exciting any venereal affection in the deep-seated parts; and our author introduces a general remark, that, for all *scaly eruptions*, at whatever period of the disease they may occur, mercury may be given with every prospect of advantage. In selecting the form of mercurial preparation, our author's practice is to exhibit the solution of muriate of mercury, when the skin and throat are affected; if the patient be particularly delicate, either the compound calomel pill, with the decoction of sarsaparilla, or five grains of the blue-pill every night; but, in the event of the bones or other deep-seated parts being attacked, where it may be necessary to excite a full mercurial action, mercurial frictions are often preferable.

With respect to the treatment of the sore-throat, we shall give Mr. Carmichael's direction in his own words; simply because they contain one of those numerous avowals of the discordance of his theory and practice, which are so frequently to be met with throughout the volume. We shall draw no inferences from the passage; they will be sufficiently obvious to every one who attentively peruses it.

"If the ulceration is of small extent, representing a white apthous looking surface, any of the following local applications may be employed every third or fourth hour, by means of lint on the end of a probe, or a large camel's-hair pencil,—viz. oxymel æruginis, or a solution of the nitrate of silver, in the proportion of from six to ten grains to an ounce of distilled water, or a solution of the oxymuriate of mercury, in the proportion of from three to six grains to an ounce of distilled water.

"But, if the ulceration is extensive, engaging the entire fauces as far as can be inspected, and having perhaps already destroyed the uvula, and a great portion of the velum, recourse should immediately be had to fumigations of the red sulphuret of mercury (factitious cinnabar) employed every fourth or sixth hour; or, if the patient cannot bear the fumes of this preparation, fumigations of the hydrargyrus cum creta may answer equally well. It will be objected to me, that the adoption of these means is in direct opposition to the preceding doctrine, that mercury ought not to be employed until the disease is on the wane: whereas, it is here recommended in a way which often mercurially affects the constitution, while the poison is in its state of highest activity. This is undoubtedly true; but my answer is simply, that it is always better, of two evils, to chose the least; and, therefore, that it is more prudent to adopt the use of a remedy capable of checking a dangerous ulceration, even though that remedy, by exciting general mercurial action, may render the disease, of which the ulcer of the throat is but a symptom, more unmanageable, than to permit a malignant ulcer to proceed in destroying parts of undoubted importance, and which, perhaps, involve life itself in their destruction.

"Fumigations of sulphuret of mercury may, however, only act locally on the sore, without mercurially affecting the constitution; and I make it a rule, as soon as I perceive any amendment in the state of the ulcer, to discontinue them, in order to prevent, as far as is consistent with the use of their application, the constitution from becoming mercurially affected: and thus we may receive all the benefits, and escape the dangers, which the remedy is capable of effecting.

"So great is the utility of those applications, that, in the numerous cases of extensive ulceration of the pharynx which have come under my observation, I do not recollect one which did not yield to their influence, unless the ulcer had extended upwards into the nares, or downwards into the larynx. When either of these misfortunes has occurred to the patient, his situation is truly critical; for parts have now become affected, which, from their structure and functions, are not easily remedied; and it requires great prudence and circumspection in the practitioner to decide upon the means to be employed, and which may be pursued with steadiness; as nothing is more frequently injurious than vacillation." (P. 204—206.)

The remaining part of this long chapter is filled with cases illustrating the evils produced by the too-early exhibition of mercury in this form of disease; and others, proving its beneficial operation when given at the proper period; and these interesting details are followed by many pages of controversy, involving, in fact, the whole of our author's peculiar opinions, —arguments which we have not space to enter into, and which we regret the less, since the question between him and his opponents is not one that arguments ever will settle; it is one of *facts* merely: and those facts, as far as our experience goes, (and we are borne out in this remark by every surgeon with whom we have conversed with on the subject,) are decidedly against his arrangement and theory. Whilst in many points of his practice we have great pleasure in agreeing, and stating our unfeigned belief that they constitute a very important improvement in the treatment of the venereal disease, we conceive that it cannot be too often repeated, that, whenever there is much pain in any ulcer, whenever there is constitutional disturbance or fever, whatever the part affected may be, there the internal exhibition of mercury is pernicious; but, beyond that, we do not pursue the same road as our author. We do not hesitate to give mercury to all rebellious sores, not included in the above description; and we do not hesitate to say that, by so doing, we reduce the chances of secondary symptoms to a mere nothing: but we must not give this remedy without precaution, without strict confinement, and without attentively watching its effects; for, if these points are neglected, we conceive it to be much safer to let the disease run its course.

We have now arrived at the sixth chapter, containing an account of the *Scaly Venereal Disease;* in fact, the only form which he acknowledges to be syphilitic. He thus describes the disease:—

"The primary syphilitic ulcer, or that which gives origin to the scaly eruption, has been well described by Mr. Hunter in the following words: 'The sore is somewhat of a circular form, excavated, without granulations, with matter adhering to the surface, and with a thickened edge and base. This hardness, or thickening, is very circumscribed; not diffusing itself gradually and imperceptibly into the surrounding parts, but terminating rather abruptly.'

"Every word of this description should be strictly attended to, as conveying an exact definition of chancre; so far, at least, as it occurs on the glans and prepuce, though not on the body of the penis, where a slight difference is observable. Ulcers which are not syphilitic may, but seldom have a fulness and slight induration round the circumference; but, then, this induration does not convey that sensation of solidity and firmness to the touch which that of a real chancre possesses; neither does it terminate abruptly, but diffuses itself gradually and imperceptibly into the surrounding parts; in which circumstance it differs from chancre so evidently, as to be at once distinguished by an experienced practitioner.

"The induration of a chancre is not confined to the edges only, but extends under the entire surface of the ulcer. We often meet with chancres in which the ulceration is inconsiderable, when compared to the extent of the induration; and even instances of an indurated knob, or tubercle, on the penis, without any visible ulcer, which have been followed by the constitutional symptoms of syphilis, are not uncommon: but, on inquiry, we shall probably learn that, in every such instance, a small ulcer existed at first on the callous part, which healed under the use of some local applications. Chancre is, in fact, an indolent ulcer, and makes but slow progress, compared to those ulcers of the parts of generation which are destitute of any surrounding induration, and particularly the phagedenic and sloughing ulcers." (P. 304, 305.)

We are then told to recollect that frequent irritation is capable of producing, around the most simple sore, considerable induration; and experiments have been made, which are stated (our author prints these words in italics,) to have produced so great a degree of callosity, as to deceive the most experienced practitioner. This Mr. C. doubts, as well he might, since it is fatal to his diagnostic mark; but it is nevertheless true.

Chancre, then, (for to this form of ulcer our author limits this term,) is of a dark livid colour; but it is not excavated, and is generally from the size of a sixpence to that of half-a-crown. Mercury soon produces a healthy appearance in this sore; but, unless it be given, its livid surface is alternated, every three or four days, with that of a light brown or tawny colour; the ulcer spreads slowly, and, as it spreads, the induration increases.

We pass by a few remarks on *bubo*, and proceed to the constitutional symptoms following this ulcer. Here our author expresses his belief that ulceration of the throat is frequently the first intimation we have of the disease becoming general; but the patient's health commonly declines previously to the appearance of the eruption, which is always scaly, and for an accurate description of which he refers to Dr. WILLAN,* who has described two species. This eruption is, in almost every instance, to be found on the forehead, breast, back of the neck, groins, and adjoining surface of the pubes. On the back of the neck, and near those parts covered with hair, the spots spread into each other, so as to form extensive copper-coloured blotches. When the eruption affects skin which is opposed to skin, as between the nates, or the scrotum and thigh, &c. it is not scaly, but the skin becomes elevated into a moist, soft, sometimes convex, surface, which discharges a whitish matter, and to which our author believes the terms of fici, cristæ mariscæ, &c. have been applied. If mercury is not employed, the eruption proceeds to ulceration: thus, each spot is covered by scales or by scurf, which is thrown off and succeeded by another, each being thicker than the preceding, till at length it forms a crust, under which matter collects, and it becomes a true ulcer.

The ulceration of the throat is seated in the tonsils; is not accompanied by much pain or swelling, and produces a considerable excavation of the tonsil. The bones, periosteum, fasciæ, and ligaments, are the deep-seated parts most liable to the attacks of the syphilitic poison. Of the bones, those nearest the surface are most usually affected. Swellings of the testes, tendons, or fasciæ, are generally indolent, and do not excite any pain. The true node is a solid enlargement of the bone, which is indolent in its commencement. The true syphilitic pains are supposed to affect the centre of the long bones, though this is not universally the case. For all these symptoms, mercury is the certain cure; and our author remarks the certainty and rapidity with which all the symptoms affecting the soft parts yield to its employment.

There is in the treatment of this disease, he observes, nothing perplexing or dubious, if we except the mercurial phagedena, and other effects of that mineral. To these effects some pages are devoted, to which we can only allude; and this leads us to Mr. Carmichael's avowal, that this, the *true* venereal disease, is, as well as the other forms, curable without mercury; a fact which he had denied in the first edition of his work. The two cases which have led him to this conclusion are inserted, upon which he makes the following remarks:—

"Although these two cases cannot fail to make a due impression, yet, if they stood alone, their evidence could not be deemed sufficient to establish a belief that true syphilis, like the papular disease, is capable of yielding to the powers of the constitution, or to remedies in which mercury does not form an ingredient. But this deficiency is

---

* Page 169.

amply supplied by the testimony of Messrs. Guthrie, Rose, Hennen, Dr. Thomson, and other equally intelligent surgeons.

"It must be admitted, however, from these cases, that, although mercury may not be absolutely necessary for the cure of true syphilis, yet that recovery may be greatly expedited by the exhibition of that medicine.

"In thus relinquishing my opinion that true syphilis differs from other venereal complaints, by always requiring mercury for its cure, it is necessary to reduce the doctrine I hold to this proposition,—that, with respect to the use of that medicine, it differs from them only in not being injured, but decidedly benefited, by it, in *all* its symptoms and stages. I may not soon have an opportunity of advancing this investigation much further, on account of the extraordinary fact already adverted to, that syphilis is now comparatively seldom to be found in this country, although the very form of venereal disease which, there is reason to believe, was most predominant in the time of Hunter." (P. 342, 343.)

From the last chapter, containing remarks upon diseases most likely to be confounded with those of venereal origin, we shall not make any extracts. Neither have we space to insert our author's synopsis of the work; though, on some future occasion, we shall take an opportunity of presenting it to the consideration of our readers.

We here take our leave of Mr. Carmichael. Though we differ from him, *toto cœlo*, in all that regards his theoretical views, and in every thing that relates to his multiplication of venereal poisons;—though we are firmly persuaded that the proscription of mercury is too general, and has been the cause, not in his hands only, but in those of the non-mercurialists on this side of the water, of an infinite proportion of secondary cases, that never used to occur, and never would have occurred, had not the recent inquiries been carried too far, and been suffered to influence our practice too extensively;—though we regret his arrangement of primary ulcers, which he might as well have divided into twenty or fifty species, as into four or five, —yet, with all these errors, for such we believe them to be, (whether ignorantly or not, time only can show,) there is much excellent advice in the work relative to the local and general treatment of the disease. His chapter on the phagedenic sore is very valuable; his cases are highly interesting, and well detailed; and no one ought to be satisfied without perusing the volume itself.

We had intended to have added some remarks upon the non-mercurial practice generally, but we have so far exceeded the limits assigned to us, that we must either postpone the intention for the present, or perhaps abandon it altogether; though that will very much depend upon what is said or done upon this subject in other quarters; and it is of great moment that the profession should come to a right understanding on a point of such importance as the treatment of a disease, which, if mismanaged, not only entails misery upon the immediate sufferer, but affects the welfare of generations yet unborn.

---

### DIVISION II.

### FOREIGN.

ART. IV.—*Manuel d'Anatomie Générale, Descriptive et Pathologique;* par J. F. MECKEL, Professeur d'Anatomie à l'Université de Halle. *Traduit de l'Allemand, et augmenté des Faits nouveaux dont la Science s'est enrichie jusqu'à ce jour;* par A. J. L. JOURDAN, Membre des Académies Royales de Médecine de Paris, &c. et G. BRESCHET, Professeur agrégè en Exercice, Chef des travaux Anatomiques de la Faculté de Médecine de Paris, &c. Tome troisième.—Paris: Baillière, 1825.

[Concluded from page 434.]

WE resume our analysis of M. MECKEL's work, with the subject of *the Peculiarities of Structure of the Glandular System.*

The substance of glands is not regenerated, when it has once been destroyed; but they are subject to a great number of anomalies in their original formation, such are as follow:—They may be totally or partially wanting: the partial absence is principally witnessed in those which exist in pairs, as the kidneys and testicles; the glands connected with the sexual organs are those most frequently entirely absent. Smallness is less common, although all the glands furnish occasional examples of it: some are more especially liable to this deformity, such as those connected with generation. Increased number of glands is, for the most part, only apparent, arising from parts, which in the natural state are simple, being divided. Excess of volume is rarely congenital: however, the liver and thyroid gland are occasionally found, at birth, to be much larger than natural. Under this head might be ranged certain glands retaining permanently that great degree of development which characterises them in the early part of life, particularly the liver and thymus gland. The division of a simple gland into many, is principally witnessed in the spleen; more rarely in the thymus, thyroid, kidneys, and liver. Of unnatural union, the kidneys furnish the most frequent example.

Unnatural situation is much more frequently illustrated by some glands than others: the kidneys, testicles, and liver, vary much more than any other in this respect. The kind of anomaly with regard to situation is not the same in all the glands; the kidneys may be placed extremely low down; the testicles high up; the liver without the cavity of the abdomen, &c. The non-symmetrical glands are subject to inversion: thus, we sometimes have the liver on the left side, and the spleen on the right.

The accidental deviations from natural conformation, which may supervene in the glands at any period of life, are of the same kind as the congenital. It is not uncommon to see them diminished or wasted: this happens frequently with the liver, spleen, testicle, supra-renal capsules, &c.; but in general this diminution is accompanied by alteration of sensible structure, to a greater or less extent.

The glands increase in size much more frequently; and this is either primitive, or the result of other organs becoming inactive. In this case, likewise, the texture often, but not always, suffers some modification; of which, the slightest degree consists in the change of colour, and the increase or diminution of consistence,—more frequently the former; in a higher degree, the increase depends upon the effusion of serum, blood, or fibrine; and, in a degree still greater, new and unnatural formations of various kinds, are developed. Notwithstanding, the increase of volume is sometimes pure and simple, particularly when one organ takes upon itself the functions of its fellow,—as in the kidney, when one only is left. The liver, likewise, is frequently enlarged after ague, without any other alteration of structure; but, in the other glands, this phenomenon is rare.

The accidental displacements are uncommon, on account of the strength of the bands which attach the glands: occasionally, however, they are witnessed, as in hernia.

The increase and diminution of consistence pave the way, from vices of formation to changes of texture. The glands are often too hard; too soft; of a tissue too lax, and easily torn: indeed, in many instances, no other change is perceptible. When the structure has become too soft, they are very liable to suffer lacerations; of which, the liver and spleen afford numerous examples.

The alterations of texture in glands are very numerous, depending, no doubt, upon their functions, which consist in the formation of some substance different from their own. These alterations consist, for the most part, in the development of a particular solid in the middle of the gland, while the secreted fluid is voided in the natural state. With respect to the frequency of their changes of texture, and the resemblance of these to natural structures, the glands might be enumerated nearly in the following order, *inversely*,—the mammary glands, supra-renal capsules, spleen, testicles, mucous glands, lymphatic glands, liver, thyroid, and ovaries. These last are unquestionably subject to the most frequent degenerations, and produce the substances most resembling structures which are natural,—such as serous or mucous membranes, cartilage, bone, fat, hair, teeth, &c.

Inflammation of the glands rarely ends in mortification; more generally in exudation, which is the principal cause of the hardening of glandular structures, and in suppuration, which sometimes completely destroys the texture. These different peculiarities appear to depend upon the functions which the glands perform.

It does not appear that glandular structure is ever an accidental development, owing to the great complexity and peculiar characters presented in its formation.

### *Accidental Formations.*

Accidental formations are sometimes produced from a particular fluid, which is poured out expressly to give rise to them; sometimes they are formed by precipitation and crystallisation, after the laws of chemical affinity, in a secreted fluid, the composition of which has undergone some change. The second of these modes of development occurs in calculi. The first belongs to all other accidental formations, whether they have or have not primarily, organic connexions with parts already exciting. All changes of texture have for their proximate cause some anomaly in the nutrition. However different may be the products of this function, when deranged, they resemble each other very much with respect to their origin; and, with regard to many, the differences which they present, after the lapse of a certain time, depend either upon the nature of the parts in the interior or neighbourhood of which they are formed, or upon external and accidental circumstances. All alterations of texture proceed from an albuminous substance, which probably is always fluid at the moment of its effusion.

Dropsy, that is to say, the unnatural accumulation of albuminous fluids, may be regarded as the first step which is made towards the formation of new substances. Sometimes these fluids are clear and limpid; often they contain coagulated flocculi. They vary very much with respect to their specific gravity, their degree of coagulability, and the respective proportions of their constituent ingredients, whether fluid or solid,—especially with regard to the quantity of albumen which they contain. The proportion of salts, on the contrary, is said to be nearly the same in all; an assertion which agrees with the observations of Drs. MARCET and BOSTOCK.* Acids and heat

* *Medico-Chir. Transactions*, vols. ii. and v.

easily coagulate the serosity in ascites, dropsy of the pericardium, hydrothorax, hydrocele, &c., which contain much albumen. That of hydrorachitis and hydrocephalus experience little change, on account of the small quantity of albumen they contain.

It frequently happens that, in consequence of the coagulation of the effused fluids, adhesions take place between organs which remain isolated in their natural state,—such as those enveloped by a serous membrane. When the coagulability attains a certain degree, or a portion of the fluid has been removed, the parts are merely adherent; but when the quantity of fluid poured out has been considerable, the organs are not only adherent, but, as it were, enveloped in a thick layer of coagulated substance, so that they can no longer be distinguished from each other. The alteration of nutrition which produces this intimate union, is more severe than that which merely produces accumulation of fluids: it constitutes the state called *inflammation*.*

The quantity and consistence of the uniting substance varies much; it is not uncommon for it to become ossified. This is the origin of the greater number of ossifications, particularly those which occur between the corresponding surfaces of serous membranes, whether they assume the form of scales, or the less frequent appearance of filaments. The organs surrounded by this coagulation are generally sound; or, at least, if they present some slight changes, they are only such as are attributable to compression.

Formations which are very solid, white, and in which scarcely a trace of any texture is to be distinguished, have the general name of Steatoma, or that of Sarcoma. It is extremely probable that all tumors have this form at their commencement: analysis shows that they are entirely composed of albumen.

In general, unnatural productions adhere, in a greater or less degree to the parts, in the vicinity of which they are developed. This, however, is not the case with regard to all; for they are occasionally met with quite free: such, in particular,

* Meckel regards inflammation, or an analogous action, as the principal means by which all formations take place, whether they be regular or irregular, —that is to say, all the accidental tissues, with or without their analogies in organisation; and he places the seat of inflammation in the sanguiferous capillaries. Broussais, in his History of Chronic Inflammations, (Paris 1808,) enumerates as the effects of inflammation—pain, tumefaction, redness, heat, gangrene, hardening with redness, suppuration, ulceration, development of false membranes, white and tubercular suppuration, fatty indurations, cancer, dropsy, &c. In another work, (*Examen*, Paris 1821,) granulations, whether cartilaginous, bony, or calcareous, melanosis, schirrus, and tubercles, are enumerated as the results of inflammation. But, according to him, in order that most, if not all, of these alterations may take place, it is necessary that inflammation of the white vessels of the part be joined to that of the red. It is to this that he gives the name of sub-inflammation;—this which he regards as entering into all chronic inflammations, and producing the chronic alterations of structure.—(*Note by the Translator.*)

are the secretions of fibrine which form in the vascular system, and likewise many cartilaginous and bony substances which are met with in the cavity of serous membranes, calculi, and intestinal worms. Many of these substances have connexions with the neighbouring parts at first, and are, in truth, excrescences from them. Calculi and intestinal worms do not come under this class; for it cannot be doubted that they are formed in, at the expence of, a fluid.

In examining with care the relations between the organs and these alterations of texture, we perceive that they are the result of the conversion of the natural substance of the former into some other texture, more or less different; or that they form in the organ, either from within outwards or from without inwards, a foreign body, which either destroys the organ, to a greater or less extent, by a mechanical influence, or occasions in it an increase of volume. This difference, however, is perhaps more apparent than real; at least, it only refers to the exterior,—that is, the form. In fact, in the second case, the accidental substance is uniformly mixed with the natural, or this last is more or less converted into it. In the first, it forms distinct and isolated depositions; but the essence of this state consists in the production of a substance different from the natural tissue, as the consequence of a change, derangement, or anomaly of the nutrition, under whatever form this new product may present itself, even when it is enclosed in a cyst which separates it from the natural structures; whether this cyst has appeared first, and secreted its contents, or its formation be consecutive, and purely accidental.

All alterations of structure are, therefore, to be considered as new formations, which sometimes are repetitions of those naturally existing in the body, and at others are quite different from natural parts. Even these last, however, have some analogy with natural tissues; on which account they were long called by names borrowed from them,—names which some moderns still retain: but, the analogy being often very loose, we are not justified in regarding all new and morbid formations as repetitions of what is natural. This, however, has lately been done by KEISCHMAN, who opposes sarcoma to muscle,—steato-sarcoma to fat and muscular fibre,—pancreatic sarcoma to the pancreas,—and medullary sarcoma to the brain. M. DUMAS likewise considered all the morbid alterations of organs as transformations of these organs into each other. This hypothesis, indeed, which is justly objected to by Meckel, has long been expressed in the different names given to tumors, and by the theories proposed to explain their formation.*

---

* Vide PLENK *de Tumoribus*, Vienna 1767.

The history of formations of the first kind is given along with those of the regular tissues, of which they are repetitions; but there is only an almost insensible shade of difference between the phenomena of their production and the regeneration of organs; particularly of some of them, as the bones. Both sorts of accidental structure very often exist together; as indeed, in general, the simultaneous occurrence of different unnatural productions is very common. A certain degree of regularity appears to take place in this respect; for there are certain accidental formations which are more particularly associated, in which case sometimes the substances are perfectly distinct, and placed side by side; at others, they are divided into minute portions, and so intermixed, that they appear to constitute a new and peculiar tissue.

The classification of tissues which are entirely new presents great difficulties, both on account of the uncertainty of the signs by which they may be recognised, and the gradations by which one passes into the other, as well as the modifications which they present, according to the different organs in the midst of which they are developed, and the frequency of the cases in which many new formations, entirely distinct, are found together. There are some of these tissues, concerning which it is difficult to say whether they be simple excrescences, or partial enlargements of the substance of the organ, or actually new formations; such, for example, are the polypi of mucous membranes. Nevertheless, according to Meckel, three kinds of unnatural formations may be admitted, which have with natural organs connexions similar to those existing between these last themselves,—viz. *tubercular* tissue, *schirrous* tissue, and perhaps *fungous* tissue. Besides these three, Mr. Abernethy admits *pancreatic* sarcoma, (which Meckel regards as a modification of accidental fibro-cartilaginous tissue, determined by the nature of the parts in which it is developed,) *mammary* and *medullary* sarcoma; which last resembles closely the *encephaloid* of M. Laennec. Meckel regards the *melanosis* of this last writer as differing but little from his encephaloid formations.

The tubercular tissue or scrofulous degenerations, and schirrus cancerous or carcinomatous structures, resemble each other in some points of view, and differ in others. It is in consequence of their relations with each other, and with other unnatural formations, that the names tubercle and schirrus are employed in so general a manner; and thus many pretended cancers of the uterus are no more than fibrous tumors. The characters common to these two textures are, to be more or less hard, and to have a greyish colour; their hardness is increased by the action of acids and heat: in time they become soft, and filled with fluid. They may be distinguished, however, by the following characters:—*Tubercles* are homogeneous, of a dull white, sometimes yellowish and opaque; they become converted into a white and friable mass; then into an unctuous fluid, in which are little irregular flocculi, grey, and like cheese; they present themselves under the form of rounded bodies, sometimes enclosed in a cyst, or in irregular masses, which occupy a large portion of the organ in which they are developed. *Schirrus* is composed of two substances: one white, fibrous, opaque, and which forms a net-work; the other semi-transparent, somewhat shining, bluish or greenish, more rarely white or reddish, and which is inclosed in the meshes of the other. It becomes converted into an ichorous suppuration, which corrodes the skin, and gives rise to fungous growths, and to ulcers with reverted edges.

Tubercles have their seat principally in the lungs and lymphatic glands, particularly those of the mesentery; in the mucous membrane of the intestinal canal, where, above all, they are developed in the latter stages of tubercular phthisis; more rarely in the liver, spleen, testicles, kidneys, and muscles. Schirrus prefers the glandular organs, especially the mammæ, the uterus, the prostate, the intestinal canal, and the skin, from which it extends to the lymphatic glands and neighbouring parts.

According to our author, the fungous hematodes of Hey, the spongoid inflammation of Burns, and the melanosis of Laennec, are all the same, differing from the preceding form of schirrus only in having less induration and a blackish colour. Nevertheless, they resemble it so much as to have received the name of soft cancer.

All these unnatural formations, of which we have spoken, may be regarded as integral parts of organisation, on account of their connexion with the surrounding textures. There are others, however, which have no connexion, which are entirely free, and only derive their nourishment from the neighbouring parts. These are intestinal worms, or entozoares, which live, some in particular cysts, and others in immediate contact with the substance of the organs. The lowest in the scale of these are like free vesicles, with their walls semi-transparent, rounded, perfectly homogeneous, and filled with a serous fluid; their number and size vary very much, and they are developed both in the substance of organs and in their cavities. These vesicles, Meckel thinks, ought to be regarded less as animals, than as analogous to the membranes of the egg. The common name for them is hydatids; that of M. Laennec, acephalocysts. The principal situations in which they are met with are the liver, (whence they enter the cavity of the abdomen,) the ovaries, the kidneys, brain, lungs, and testicles; but there is no organ in which they have not occasionally been found.

After the hydatids, come the vesicular worms, which are contained in particular cysts; and then the other intestinal worms, the numerous species of which we shall not enumerate, having lately had occasion to do so.

Calculi are formed chemically, by means of precipitation and crystallisation in secreted fluids: with these M. Meckel concludes his account of accidental formations, and we our extracts.

### MEDICAL AND PHYSICAL INTELLIGENCE.

